# Extracellular and intracellular small-molecule galectin-3 inhibitors

**DOI:** 10.1038/s41598-019-38497-8

**Published:** 2019-02-18

**Authors:** John Stegmayr, Fredrik Zetterberg, Michael C. Carlsson, Xiaoli Huang, Gunjan Sharma, Barbro Kahl-Knutson, Hans Schambye, Ulf J. Nilsson, Stina Oredsson, Hakon Leffler

**Affiliations:** 1 Department of Laboratory Medicine, Lund University, 22100 Lund, Sweden; 2 Galecto Biotech AB, 2200 Copenhagen N, Denmark; 3 Department of Cellular and Molecular Medicine, University of Copenhagen, 2200 Copenhagen N, Denmark; 4 Department of Biology, Lund University, 22100 Lund, Sweden; 5 Department of Chemistry, Lund University, 22100 Lund, Sweden; 6 Present Address: Agilent Technologies Denmark ApS, 2600 Glostrup, Denmark; 7 Present Address: Xintela AB, 22381 Lund, Sweden

## Abstract

Galectin-3 is a carbohydrate binding protein which has important roles in cancer and immunity. Potent galectin-3 inhibitors have been synthesized, for experimental purposes and potential clinical use. As galectin-3 is implicated in both intra- and extracellular activities, permeability of galectin-3 inhibitors is an important parameter determining biological effects. We compared the cellular uptake of galectin-3 inhibitors and their potency in the intracellular or extracellular space. The inhibitors differed in their polar surface area (PSA), but had similar affinities for galectin-3. Using a well-established permeability assay, we confirmed that the uptake was significantly higher for the inhibitor with the lowest PSA, as expected. To analyze intracellular activity of the inhibitors, we developed a novel assay based on galectin-3 accumulation around damaged intracellular vesicles. The results show striking differences between the inhibitors intracellular potency, correlating with their PSAs. To test extracellular activity of the inhibitors, we analyzed their potency to block binding of galectin-3 to cell surfaces. All inhibitors were equally able to block galectin-3 binding to cells and this was proportional to their affinity for galectin-3. These inhibitors may serve as useful tools in exploring biological roles of galectin-3 and may further our understanding of intracellular versus extracellular roles of galectin-3.

## Introduction

The galectin family of carbohydrate binding proteins have gained increasing interest as therapeutic targets in several diseases, such as chronic inflammation and cancer^1–4^. Galectins are soluble proteins synthesized on free ribosomes in the cytosol. Even though they lack the classical characteristics of secreted proteins, they are rapidly translocated to the extracellular space through a yet unknown pathway^5^. Once in the extracellular environment, the galectins are exposed to a large variety of glycan structures, where they recognize and bind specific β-galactosides. As some galectins are able to form multivalent structures or are multivalent in nature, they are able to cross-link glycoconjugates and form lattices. Formation of galectin/glycoconjugate lattices on the plasma membrane has been observed to influence the expression time, localization, and activity of several cell surface receptors, thus influencing numerous biological functions such as cell signaling, cell migration, and cell adherence^5,6^. Furthermore, galectins can quickly (within minutes) be recycled back to the inside of cells trough the endocytic pathway, regulating sorting of both soluble and membrane bound glycoconjugates^5,7^. Apart from the extracellular activities of the galectin family, mediated through glycan binding, galectins also play important roles in the intracellular compartments. Several studies have reported that galectins may influence cell signaling by interacting with signaling proteins in the cytosol, *e.g*. RAS proteins and β-catenin, and RNA splicing through binding of components of the spliceosome complex in the nucleus^8–12^. As complex glycans are not found in the intracellular compartment, these activities are most likely mediated trough protein-protein interactions. Interestingly, however, several of these reported intracellular activates are inhibited by molecules interacting with the galectin carbohydrate binding site, such as lactose^8,10,13^. Additionally, galectins play an important role at the interface between the cytosolic and intravesicular compartments by monitoring the integrity of vesicular membranes. It is now well established that several galectins (*e.g*. galectin-3 and -8 in particular) accumulate around disrupted vesicles by binding to exposed glycan structures and induce clearance of the damaged organelles by selective autophagy^14–20^. The diverse roles of the galectins on a cellular level have consequences for several physiological processes, in particular, related to immune responses and inflammation, as well as pathological conditions such as fibrosis, cancer, and heart disease^2,3,21,22^.

The role of galectins in physiological and pathophysiological processes has inspired the development of several distinct galectin inhibitors, both as novel therapeutics and as experimental tools for basic science^5^. The aim of the present study was to characterize synthetic small-molecule galectin-3 inhibitors for the latter application. For a galectin inhibitor to be successful as an experimental tool, it should display high affinity to the galectin of interest (to compete with endogenous ligands at biologically relevant concentrations), be selective for the galectin of interest (to ensure correct interpretation of experimental data), and be chemically stable in biological environments. Furthermore, as galectins are involved in activities on both sides of the plasma membrane, cellular uptake of the galectin inhibitors is an important parameter to consider for discriminating between activities originating from the intra- or extracellular compartment. In regard to affinity, specificity, and stability, several galectin inhibitors have been synthesized that display both high affinity (dissociation constant (*K*_*d*_) values in the low nano-molar range), selectivity for different galectin carbohydrate recognition domains (CRDs) (mainly for galectin-3), and have chemical structures unlikely to be targeted by enzymatic degradation^4,23–27^. However, less is known about the cellular uptake of these inhibitors and to what degree they are able to inhibit intracellular galectins when used in cell culture or *in vivo*.

In the present study, we compared cell membrane permeability of three previously published high affinity galectin-3 inhibitors and compared their potency in inhibiting galectin-3 activities, in the cytosol and at the cell surface. The results show striking differences in the cellular uptake of the inhibitors and of their intracellular potency, which correlates with their polar surface area (PSA). These inhibitors may serve as useful tools in exploring the roles of galectin-3 in both cell and tissue cultures or in animal models and, importantly, may further our understanding of intracellular *versus* extracellular roles of galectin-3.

## Results

### Affinity and cell membrane permeability of three galectin-3 inhibitors

**Figure 1:**
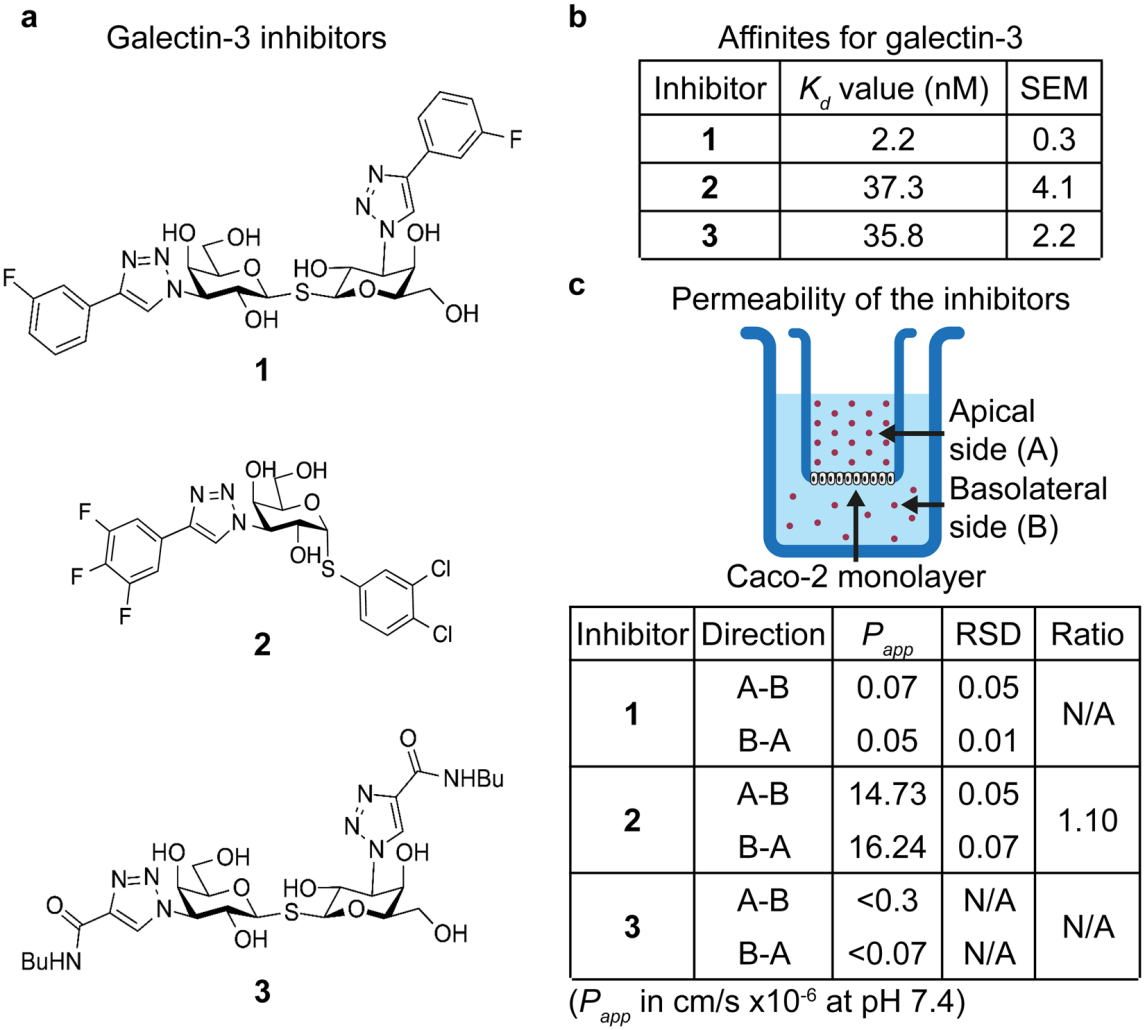
Structure, affinity, and permeabilities of the three galectin-3 inhibitors. (**a**) Structure formulas for the galectin-3 inhibitors tested in the present study. (**b**) The *K*_*d*_ values for inhibitors **1**, **2**, and **3** was obtained using a well-established fluorescence anisotropy assay. All three inhibitors displayed strong interaction with the galectin-3 CRD, with *K*_*d*_ values in the low nano-molar range, where inhibitor **1** had about a 17-folded higher affinity compared to **2** and **3**. The *K*_*d*_ values are presented as means from 9–33 measuring points (where the inhibitors generated 20–80% inhibition of the galectin-3/probe interaction) from 3 independent experiments. (**c**) The three inhibitors were tested in the well-established Caco-2 cell assay measuring the ability of compounds to cross an epithelial monolayer (in this case designed to mimic the epithelium of the small intestine). The experiments were performed at pH 7.4 and the ability of the galectin-3 inhibitors to cross the Caco-2 membrane was tested both for the apical to basolateral (A-B) and the basolateral to apical (B-A) direction, and the apparent permeability coefficients (*P*_*app*_) were calculated. Inhibitor **2** showed high permeability about equal in both directions, whereas **1** and **3** showed nearly no permeability, close to the limit of detection in the assay. RSD = relative standard deviation, ${\rm{Ratio}}=\frac{{P}_{{app}(B-A)}}{{P}_{{app}(A-B)}}$, N/A = not applicable.

Three galectin-3 inhibitors (here named **1**, **2**, and **3**) were tested in the current study, selected based on their high affinity for galectin-3 and expected differences in membrane permeability due to their polarity. Their structure, synthesis, and affinity for a wide range of galectins have previously been described in Delaine *et al*. (as compound **4**)^24^, Zetterberg *et al*. (as compound **2 h**)^27^, and Salameh *et al*. and Peterson *et al*. (as compound **47** and **29**)^23,28^, for inhibitor **1**, **2**, and **3** respectively. Inhibitor **1** and **3** are both thiodigalactosides, but with different functional groups attached to carbon 3 of each galactose residue, whereas inhibitor **2** is an α-d-galactoside with different functional groups at carbon 1 and 3 (Fig. 1a). Inhibitor **1** has shown therapeutic effects in animal models of idiopathic pulmonary fibrosis (IPF) and pathological corneal neovascularization and fibrosis^24,29^ and is now in clinical trials for IPF under the name TD139 (ClinicalTrials.gov Identifier: NCT02257177). Inhibitor **3** has recently been used in an *in vivo* study for type 2 diabetes in obese mice, in which it decreased insulin resistance and improved glucose tolerance^30^.

All three inhibitors were confirmed to have high affinities for the galectin-3 CRD, using a fluorescence anisotropy (FA)-based assay, with *K*_*d*_ values of 2, 37, and 36 nM for inhibitors **1**, **2**, and **3**, respectively (Fig. 1b). A summary of the three inhibitors affinities for the CRDs of other common human galectins (galectin-1, -2, -4N, -4C, -8N, -8C, -9N, and -9C) can be found in Figure S1a. Relative their affinity for galectin-3 inhibitor **1** and **3** also have high affinity for galectin-1, although lower compared to galectin-3. Inhibitor **2** has higher degree of specificity in regard to galectin-3 *versus* galectin-1 compared to inhibitors **1** and **3**, but instead has lower specificity when compared to galectin-4C (Fig. S1b).

The topological PSAs were calculated to be 230, 130, and 280 Å^2^ for inhibitors **1**, **2**, and **3**, respectively, according to the method described by Ertl *et al*.^31^. As the PSA of a molecule is considered to be a dominant determinant for passive cell membrane permeability and oral bioavailability (where molecules with a PSA >120–140 Å^2^ are predicted to have low membrane permeability and oral bioavailability), inhibitor **2** was suspected to have higher permeability compared to **1** and **3**^31–33^.

To test this hypothesis, we utilized a well-established assay for prediction of absorption of compounds over the intestinal epithelium, hence their ability to cross cell membranes. This assay is based on Caco-2 colon cancer cells that are seeded on a semi-permeable membrane and are allowed to form a confluent mono-layer of cells with tight junctions^34–36^. Each compound was added on either the apical or basal side of the Caco-2 cell monolayer, and its appearance on the other side was measured as a function of time (Fig. 1c). Both apical to basolateral transport (A-B) and basolateral to apical transport (B-A) was measured and the apparent permeability coefficient (*P*_*app*_) was calculated to assess active *versus* passive transport of the three galectin-3 inhibitors, where a ratio between *P*_*app*_ (B-A) and *P*_*app*_ (A-B) close to 1 indicates passive transport and a ratio below 0.5 or above 2 indicates active transport^36^. As predicted, inhibitor **2** had significantly higher values for *P*_*app*_ regardless of direction compared to **1** and **3**, suggesting higher cellular permeability of inhibitor **2** relative to the other two (Fig. 1c). The ratio for *P*_*app*_ (B-A) and *P*_*app*_ (A-B) for inhibitor **2** was 1.1 indicating that the compound is taken up through passive mechanisms (Fig. 1c). The *P*_*app*_ values calculated for inhibitor **1** and **3** were at the limit of detection, as expected for very low membrane permeability; the low values precluded the reliable calculation of the ratios between *P*_*app*_ (B-A) and *P*_*app*_ (A-B).

### Inhibition of extracellular galectin-3 correlates with the K_d_ values for the galectin-3 inhibitors

**Figure 2:**
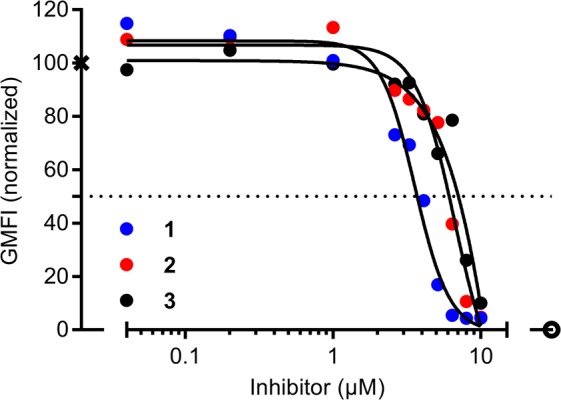
Inhibition of galectin-3 binding to the cell surface of CHO cells by the galectin-3 inhibitors. CHOZN^®^ GS^−/−^ cells were incubated 1 hour on ice with 0.5 µM fluorescein-tagged galectin-3 in the absence or presence of different concentrations of inhibitors **1**, **2**, or **3** (*x*-axis), and then analyzed by flow cytometry to determine geometric mean fluorescence intensity (GMFI, *y*-axis). The values are presented as normalized values from two experiments. The value in the absence of inhibitor was set to 100, marked by X, and the value without added galectin was set to 0, marked by open circle. The solid lines are best-fit curves.

Even though the FA assay is a valuable tool for screening galectin inhibitors in regard to their affinities for the galectin CRD, the assay does not provide information of how the galectin inhibitors behave in cellular settings, which is complicated by factors such as the increased complexity in glycan expression, the glycan linkage to proteins and lipids, cellular uptake, and degradation. Therefore, we next tested the effect of the inhibitors on galectin-3 in an extracellular setting, using an assay based on the binding of fluorescently-coupled galectin-3 (0.5 µM) to the cell surface of CHO cells at 4 °C (to prevent uptake). The binding of fluorescently-coupled galectin-3 (and other galectins) to the cell surface of CHO cells and other cell types has been confirmed to be carbohydrate-binding dependent by lactose inhibition^37–39^, lack of binding of the galectin-3 mutant R186S (with severely reduced affinity for endogenous glycans)^39,40^, and the use of genetically stable CHO cell glycosylation mutants^37,39^. In this extracellular assay, the inhibitory capacities of each inhibitor are normally directly proportional to the affinity for the galectin-3 CRD. Indeed, inhibitor **1** (highest affinity) had an inhibition curve with a lower *IC*_50_ value (~4 µM), compared to inhibitors **2** and **3** (*IC*_50_ values of 8 and 10 µM, respectively) (Fig. 2). As this assay was performed at 4 °C, to minimize endocytosis of galectin-3, we also determined the *K*_*d*_ values for the inhibitors at this temperature. As expected, the relative affinities remained the same but the absolute affinities were slightly higher for all three inhibitors compared to room temperature (Fig. S1c).

### Assay for intracellular activity of galectin-3 inhibitors

We next wanted to evaluate the intracellular activity of the galectin inhibitors. For this purpose, we sought after a galectin-3-related and carbohydrate-binding dependent intracellular process that would not be affected by inhibiting galectin-3 at the extracellular side of the plasma membrane. The rapid accumulation of galectins around disrupted intracellular vesicles offers such an opportunity. This phenomenon was first discovered for phagosomes disrupted by the cytosolic pathogen *Shigella*^14^. Galectin-3 accumulated around *Shigella* containing phagosomes within minutes after bacterial uptake into the cells. Using appropriate bacteria, cell lines, and galectin mutants, it was demonstrated that galectin-3 accumulation required the bacterial membrane disrupting machinery, the glycan binding activity of galectin-3, and host cell glycoproteins with galactose containing N-glycans^14^. Thus, it appears as if upon vesicle membrane disruption, intravesicular host glycans become exposed to cytosolic galectins that rapidly accumulate at the location. This has since been shown for other galectins and for other ways of disrupting vesicular membranes (*e.g*. chemical treatment) and galectin-3 has furthermore been established as a marker for vesicle damage in a variety of studies^15–19,41^.

With this in mind, we speculated that a galectin inhibitor that is able to pass the plasma membrane and retain its activity would be able to inhibit the carbohydrate binding-dependent galectin accumulation upon vesicle insult. In order to establish a simple system which could be standardized, we chose to use treatment with glycyl-l-phenylalanine 2-naphthylamide (GPN), which is a well-established way to chemically induce vesicle damage. GPN is a lysosomotropic peptide that quickly accumulates inside lysosomes where it is hydrolyzed by the lysosomal enzyme cathepsin C, which results in the formation of charged amino acids that are trapped in the vesicle causing osmotic lysis^42,43^. Additionally, GPN has been shown to induce galectin-1, -3, -8, and -9 accumulation around damaged lysosomes in HeLa cells following treatment with 0.3 mM GPN for 10 minutes^15^.

**Figure 3:**
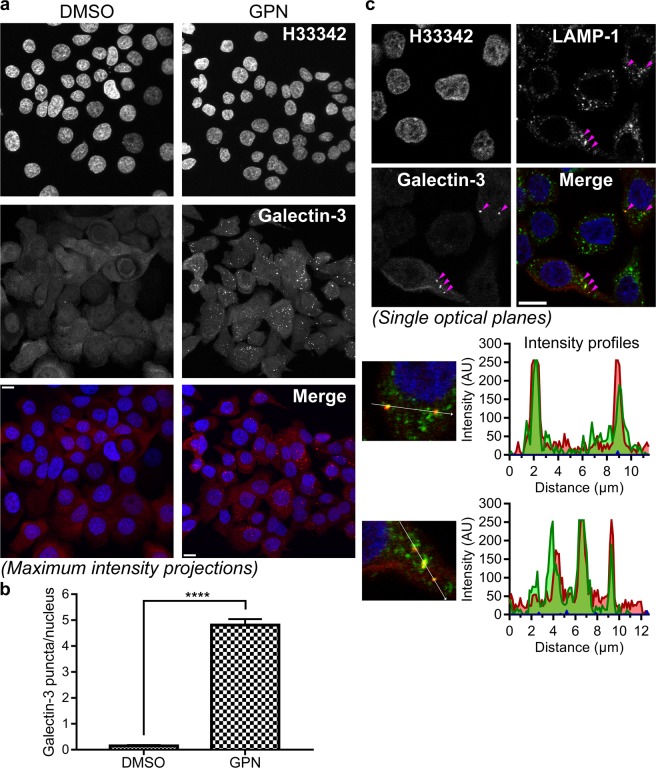
Rapid accumulation of galectin-3 around lysosomes upon chemically-induced lysosomal damage. (**a**) JIMT-1 breast cancer cells were treated with 0.3 mM glycyl-l-phenylalanine 2-naphthylamide (GPN) or 1% (v/v) dimethyl sulfoxide (DMSO) (used as control) for 13 minutes. The samples were then fixed, permeabilized, and stained to visualize nuclei (H33342) and galectin-3. Images are shown as maximum intensity projections of *z*-stacks obtained using confocal microscopy. Treatment with GPN induces distinct galectin-3 puncta in the cytosolic space of the cells. Scale bars are equal to 10 µm. (**b**) The mean number of galectin-3 puncta/nucleus from 74 or 92 images of cells treated with DMSO or GPN, respectively, from a total of 20 independent cell cultures for each treatment. The analysis was carried out in ImageJ on maximum intensity projections of *z*-stacks. The result is presented as mean values ± SEM, and the difference was statistically evaluated by an unpaired two-tailed *t*-test (****P < 0.0001). (**c**) JIMT-1 breast cancer cell treated with 0.3 mM GPN for 13 minutes and then stained to visualize lysosomal-associated membrane protein (LAMP)-1 and galectin-3. The images (single optical planes) show clear co-localization of galectin-3 accumulations with LAMP-1, seen best by observing the intensity profiles for the vectors highlighted in white, indicating that the galectins act in the vicinity of GPN-damaged lysosomes. In addition, LAMP-1 is present in many undamaged lysosomes, without galectin-3 accumulation. The images shown are representative of a larger number of images showing similar co-localization patterns. *Arrowheads* indicate areas of co-localization and have the same *xy*-coordinates in all images. Scale bars are equal to 10 µm.

Treatment with medium containing 0.3 mM GPN for 13 minutes induced significant accumulation of galectin-3 into puncta in the cytosolic space in several cell lines, compared to cells treated with control solution (1% dimethyl sulfoxide (DMSO)) (Fig. 3a and S2). The JIMT-1 breast cancer cell line was chosen for the remaining experiments due its growth pattern (*i.e*. the cells do not grow tightly together in patches or on top of each other) and due to the relatively flat morphology of the cells, making galectin-3 puncta easier to quantify. The total number of galectin-3 puncta and nuclei in the microscopy images were quantified using the software ImageJ. The mean number of galectin-3 puncta/nucleus, from multiple fields of vision, was 4.81 ± 0.23 (n = 92) and 0.15 ± 0.04 (n = 72), after treatment with 0.3 mM GPN or 1% DMSO, respectively (Fig. 3b). Co-localization of the large majority of GPN-induced galectin-3 puncta with lysosomal-associated membrane protein 1 (LAMP-1) indicated that the galectin-3 accumulation, as expected was around damaged lysosomes (Fig. 3c).

### Inhibition of GPN-induced galectin-3 accumulation correlates with cell membrane permeability of the galectin inhibitors

**Figure 4:**
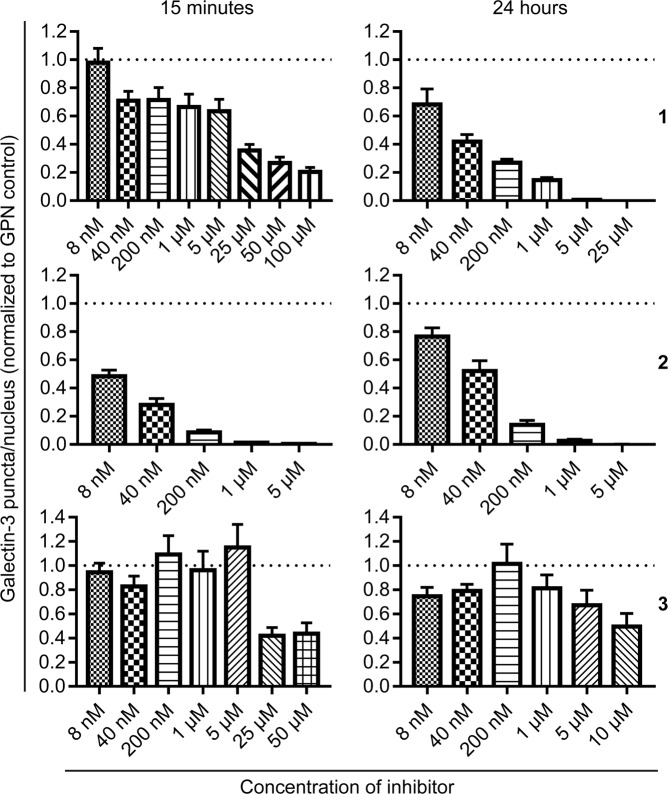
Inhibition of GPN-induced galectin-3 accumulation in JIMT-1 breast cancer cells by the galectin-3 inhibitors. The cells were pre-treated with the indicated concentrations of inhibitors for 15 minutes or 24 hours, whereupon the cells were treated with 0.3 mM glycyl-l-phenylalanine 2-naphthylamide (GPN) for 13 minutes. The number of galectin-3 puncta/nucleus was quantified for each inhibitor concentration and time point, following immunocytochemical staining and confocal imaging, and are presented relative to the number of galectin-3 puncta/nucleus in cells treated with GPN only (to better compare data set from independent experiments). Inhibitor **2** shows significantly higher inhibition of GPN-induced galectin-3 accumulation compared to both inhibitors **1** and **3** at 15-minute pre-treatment, whereas the dose-response for **1** and **2** are more alike after 24-hour pre-treatment. The results are presented as mean values ± SEM, from analysis of 7–32 confocal images from 2–8 independent cell cultures for each data set.

In the next set of experiments, we tested the potency of the three inhibitors to interfere with GPN-induced galectin-3 accumulation. When the JIMT-1 cells were pre-treated with inhibitor **2** for 15 minutes, before treatment with GPN, a clear dose-response relationship could be observed and an *IC*_50_ value of 36 ± 12 nM could be calculated (Fig. 4). Inhibitors **1** and **3** had less clear dose-response relationships, for 15 minutes of pre-treatment, and complete inhibition of galectin-3 accumulation could not be reached even at high concentrations of **1** and **3** (50–100 µM), unlike the nearly complete inhibition of galectin-3 accumulation seen with inhibitor **2** at 1 µM (Fig. 4). However, when the JIMT-1 cells were pre-treated with the different inhibitors for 24 hours, a clear dose-response could be seen for both **1** and **2** with *IC*_50_ values of 73 ± 35 and 54 ± 20 nM, respectively (Fig. 4). Inhibitor **3** was still a poor inhibitor of galectin-3 accumulation even after 24 hours of pre-treatment; although the number of puncta/nucleus were lower compared to the 15-minute pre-incubation; the nearly complete inhibition now seen for both **1** and **2**, at around 1–5 µM, could not be seen for the highest concentration of **3** (10 µM) (Fig. 4). As massive damage of lysosomes potentially could lower the pH in the cytosol or at least in the microenvironment of the damaged organelles (due to proton leakage) and due to previous findings that the specificity of galectin-3 for glycan structure is modulated by variations in pH^44–46^, we also established the *K*_*d*_ values of the three inhibitors at pH 5, 6, 7, and 8. Results show that the affinities of the inhibitors were equally affected by changes in pH, with *K*_*d*_ values of 100, 831, and 778 nM for inhibitor **1**, **2**, and **3**, respectively, at pH 5 (Fig. S1d). This shows that the different efficacies were not simply due to selective shifts in inhibitor affinities for galectin-3 with decreasing pH.

### Low basal toxicity for the galectin-3 inhibitors

**Figure 5:**
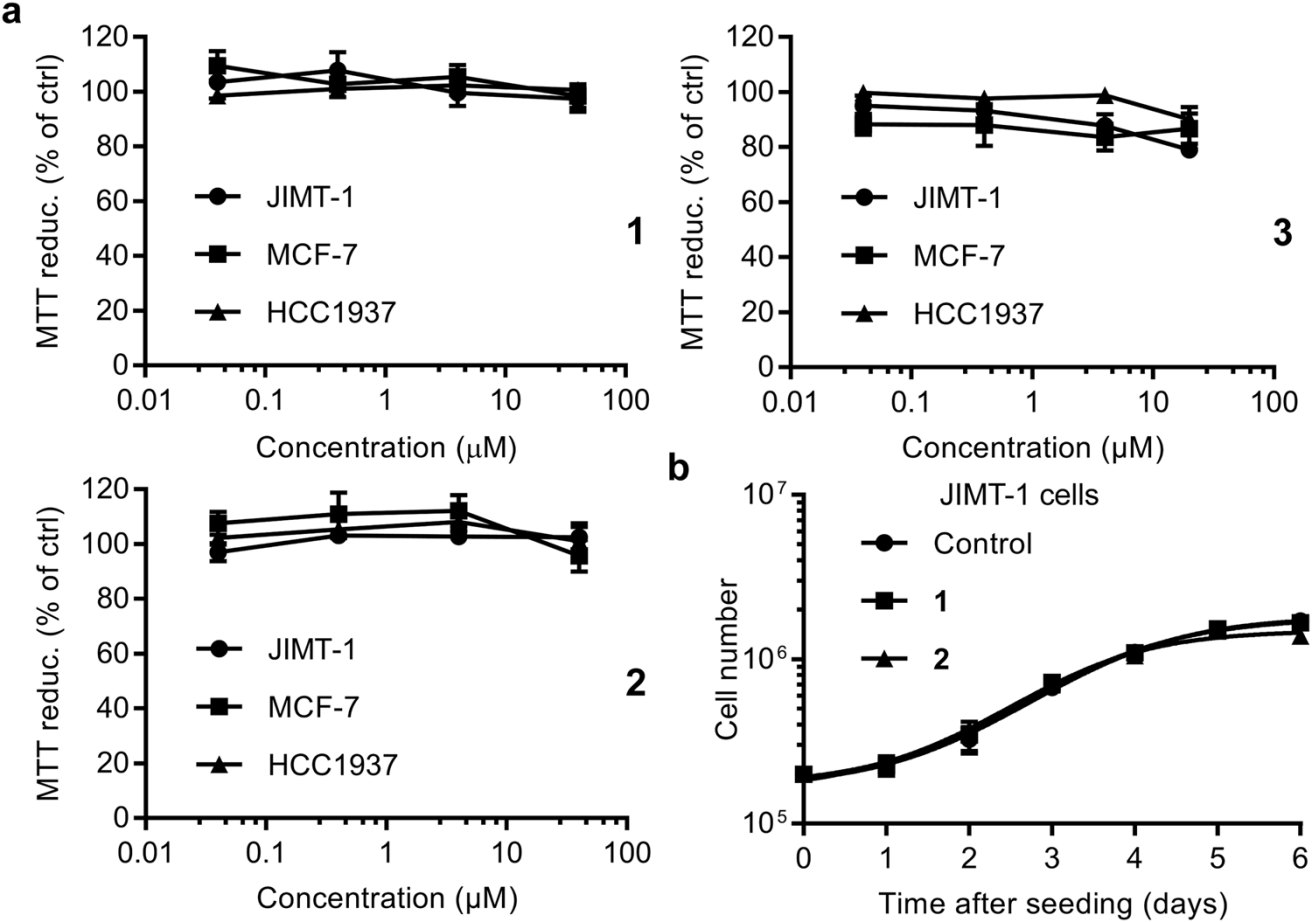
Low basal toxicity of the galectin inhibitors in two cell proliferation assays. (**a**) Three breast cancer cell lines (JIMT-1, MCF-7, and HCC1937) were cultured for 72 hours in the presence of different concentrations (*x*-axis) of the three inhibitors, and their viability measured by an MTT assay and displayed as % of the MTT reduction of control (*y*-axis). No significant effect was found for any of the three inhibitors in any of the cell lines. (**b**) Growth curves for JIMT-1 cells treated with fixed concentrations (5 µM) of inhibitor **1** and **2** or without inhibitor (control). The inhibitors were added to the cell cultures 24 hours after seeding the cells, and cell numbers (*y*-axis) determined daily for up to 6 days (*x*-axis). No effect on cell proliferation, by treatment with **1** or **2**, could be observed when comparing cells grown in the absence of inhibitor. Results in both panel a and b are presented as mean values ± SEM (n = 6).

As the inhibitors are intended to be used as experimental tools in for example cell culture and *in vivo*, we next evaluated the basal toxicity of the three galectin-3 inhibitors by measuring cell viability (MTT assay) in absence or presence of the inhibitors after 72 hours. We observed no apparent change in cell viability after treatment with any of the three inhibitors at the concentrations tested (0.04–40 µM for **1** and **2**, and 0.04–20 µM for **3**), in all three breast cancer cell lines, indicating low basal toxicity (Fig. 5a). Inhibitor **1** was, additionally, shown to have the same low toxicity in four other cancer cell lines and one normal-like breast cell line (Fig. S3a). To further validate the results obtained from the cell viability assay, growth curves were constructed for JIMT-1 cells when cultured in the absence or presence of inhibitors **1** or **2** (5 µM). As suspected, no effect on cell proliferation could be observed for either compound (Fig. 5b). Additional growth curves, constructed for inhibitor **1** (10 µM) in other cell lines also showed no effect (Fig. S3b).

## Discussion

The interest of galectins in disease continues to rise and parallel to this, several promising synthetic small-molecule galectin inhibitors (mainly targeting galectin-3) have been developed with both clinical and research implications. In the present study, we evaluated three previously published galectin-3 inhibitors for their cellular uptake and potency to inhibit galectin-3 in both the extra- and intracellular compartments. We show that the inhibitors had significantly different uptake profiles, where the inhibitors displayed both high, medium, and low cell membrane permeability, which seemingly correlates with their PSA. This, to our knowledge, is the first time a galectin-3 inhibitor with high cellular uptake is presented in the literature. These inhibitors may serve as excellent experimental tools in the quest to establish a unifying picture of the diverse roles played by galectins on both sides of the plasma membrane.

Furthermore, we present a novel assay for evaluation of galectin inhibition in intracellular environments. By treating cells with the lysosomotropic agent GPN, we induced galectin-3 accumulation around damaged lysosomes in the cytosol. This phenomenon, known to be dependent on galectin-carbohydrate interaction^14,19^, could then be used as a measurable endpoint for inhibition of galectin carbohydrate-binding activity by small-molecule antagonists in the cytosolic compartment. Comparison of three competitive galectin-3 inhibitors suggested that their potency in this assay depends on both their affinity for the galectin, their ability to cross cell membranes, and time of incubation. In an assay for extracellular activity, inhibition of fluorescein-tagged galectin-3-binding to the cell surface, their potency varied within a narrow range (*IC*_50_ values of 4, 8, and 10 μM, for inhibitors **1**, **2**, and **3**, respectively, Fig. 2) and correlated roughly with their direct binding-affinity as measured by FA (*K*_*d*_ value of 2 nM for **1**, 37 nM for **2**, and 36 nM for **3**, Fig. 1b). In contrast, in the assay for intracellular activity, **2** was much more potent (*IC*_50_ value of about 10 nM after 15 minutes pre-incubation) than **1** and **3** (*IC*_50_ values of about 10 μM, Fig. 4). This correlated with the cell permeability of the three compounds (as measured in the Caco-2 assay with **2** ≫ **1** and **3**, Fig. 1c) and the calculated PSA of the compounds, where **3** and **1** are disaccharide derivatives, while **2** is a monosaccharide derivative. Additionally, the potency of **1** in the intracellular assay greatly increased with 24-hour pre-incubation, and became similar to the potency of **2**, suggesting that it also can reach the cytosolic compartment given enough time for diffusion/transport over the plasma membrane, in accordance with what we have published previously^24,47^. Inhibitor **3** still had poor inhibitory potency even after 24 hours of pre-incubation; this may be due to its slightly higher polarity compared to **1**, and/or its >10-fold lower affinity for galectin-3. Thus, a compound’s potency in this assay reflects both its direct affinity for the target galectin-3, but also subtle difference in its ability to enter the cell. This should be useful in further development of galectin-3 inhibitors for different purposes.

Theoretically, an inhibitor might also reach damaged lysosomes within vesicular compartments in the endocytic pathway, after uptake by pinocytosis. However, this is less likely to be a major contribution to the inhibition of galectin-3 accumulation seen, because then one would have expected the potency and time course of the effect of the three compounds to be more similar.

A notable finding was also that the concentration needed to reach *IC*_50_ to inhibit cell surface binding of galectin-3 for compound **2** (8 µM) was 1000 times higher than the *IC*_50_ value in the intracellular assay (about 10 nM). This does not violate the arguments about membrane permeability described above, but requires its own explanation. In a previous study, we derived an equation that shows that with excess added galectin and high affinity cellular galectin receptors, the *IC*_50_ value of an inhibitor can be much higher than the *K*_*d*_ of its affinity with the galectin^47^. These are conditions of the cell surface-binding assay, where excess galectin was added to cells and multiple washing would have left only the highest affinity receptors for detection. In the intracellular assay, in contrast, only endogenous galectin was present and we do not know receptor affinity, degree of equilibration of inhibitor with environment, or galectin-3 concentration. Further titrations and timing in the two assays, followed by mathematical analysis is interesting for future studies, but beyond the scope of the present study.

In the present investigation, we chose to study cytosolic inhibition of galectin-3 in particular but the assay could, most likely, be applied for other galectins as well. Galectin-1, -8, and -9 also accumulate around damaged lysosomes^15^, thus the presented assay could potentially be used to investigate intracellular potency of inhibitors specific for those galectins. Here we used GPN as the lysomotropic agent, but other agents could have been used for this purpose as well, such as l-leucyl-l-leucine methyl ester^18–20^, crystalline silica (SiO_2_)^18^, and cationic amphiphilic drugs (*e.g*. terfenadine, siramesine, and amitriptyline)^19,24^. In our experimental set up, however, we were in need of a rapid induction of galectin-3 accumulation as we wanted to investigate both short and long incubation times with the inhibitors, for this reason GPN was a good option as the lysosomal damage, and galectin accumulation, is induced after just 5–10 minutes (compared to a time frame of 24 hours for cationic amphiphilic drugs)^24^.

High-affinity galectin-3 inhibitors with differences in cellular uptake opens up the possibility for interesting and novel studies, where for example galectin-3 functions associated with the extracellular environment can be more easily separated from intracellular ones (by choosing the appropriate inhibitor), aiding the quest of elucidating the biological roles of the galectin.

Interestingly, none of the galectin inhibitors (regardless of their permeability) had an effect on cell viability or proliferation in the MTT or cell growth assays (Fig. 5 and S3). This implies that galectin-3 has no effect on these cell properties, at least not under the specific conditions used in this study. It would, however, be of interest to test the different inhibitors in other cell culture systems were galectin-3 previously has been seen to influence cell proliferation. In the light of the recent finding of Thode *et al*.^48^, showing that galectin-3 (50 nM for 24 hours) induced hyperproliferation in keratinocytes, it would be interesting to investigate if treatment with the inhibitors used in this study could reduce proliferation and if the putative inhibition is dependent on the cellular location of the inhibition (*i.e*. intracellular or extracellular). This, however, is outside the scope of the present investigation, as the main purpose here was to investigate basal toxicity for the inhibitors (on- or off-target effects), as a validation for further studies.

The GPN-induced galectin-3 accumulation assay developed here, should also be useful in guiding the refining of pharmacokinetic properties of galectin inhibitors being developed as drugs. Inhibitor **1**, for example, has already been investigate in a clinical trial (ClinicalTrials.gov Identifier: NCT02257177) against IPF, but was applied topically (inhalation) since it has been previously described to have low absorption, a finding which we also confirmed in the present assay. In contrast, a compound like **2** might be available for other administration routes (*e.g*. the oral route) and may have greater tissue distribution. Inhibitor **3** was recently used in an *in vivo* model of obesity-induced insulin resistance in mice (administered by a subcutaneously implanted mini-pump), in which the inhibitor was shown to reduce the insulin resistance in both acute and chronic experiments^30^. The authors found that the major mechanism for this effect was by inhibiting the interaction of galectin-3 with the extracellular part of the insulin receptor. This hypothesis is further strengthened by the observations in the current study, that inhibitor **3** has very low cellular uptake, and thus has to exert its major effect in the extracellular compartment.

In conclusion, we characterized galectin-3 inhibitors that may be used as novel experimental tools in galectin-3-related research, based on their pronounced differences in cellular uptake. Furthermore, we developed a novel assay which could be used to screen future galectin inhibitors for intracellular activity and potentially help in the understanding of the pharmacokinetic-pharmacodynamic relationships of the compounds and for understanding the role of galectin-3 in a broad range of biologic and pathologic conditions.

## Methods

### Materials

Dulbecco’s Modified Eagle Medium (DMEM)/Ham’s F12 medium mixture (1:1), RPMI-1640 medium, fetal bovine serum (FBS), penicillin/streptomycin solution, nonessential amino acids, l-glutamine, and formaldehyde were from Merck Millipore (Billerica, MA, USA). Trypsin was from VWR International AB (Lund, Sweden). Cell culture plastic, Alexa Fluor^®^ 594-conjugated goat anti-rat IgG antibody and Alexa Fluor^®^ 488-conjugated goat anti-rabbit IgG, NHS-fluorescein and propidium iodide (PI) were from Thermo Fisher Scientific (Waltham, MA, USA). Insulin, epidermal growth factor, TPP^®^ TubeSpin bioreactor tubes, EX-CELL^®^ chemically-defined serum-free medium for CHO cells, DMEM/Ham’s F12 medium with 15 mM HEPES (2-[4-(2-hydroxyethyl) piperazin-1-yl] ethanesulfonic acid) buffer, DMSO (cell culture grade), Hoechst 33342 (bisbenzimide), Mowiol^®^ 4–88, bovine serum albumin (BSA), β-d-lactose, *N,N*-dimethylformamide and TWEEN 20 were from Sigma-Aldrich (St. Louis, MO, USA). GPN was from Santa Cruz Biotechnology, Inc. (Dallas, TX, USA). PD10 columns were from GE Healthcare (Chicago, IL, USA). Recombinant human galectins were produced in *E. Coli* BL21 Star (DE3) cells and purified by affinity chromatography on lactosyl-sepharose columns, as previously described in Salomonsson *et al*.^40^.

### Galectin-3 inhibitors

Galectin-3 inhibitors were synthesized by us as earlier described (**1**^24^ and **2**^27^ at Galecto Biotech AB and **3**^23^ at Lund University). The purity of the inhibitors was determined using liquid chromatography-mass spectrometry. The purity of inhibitors **1** and **2** was ≥95%. Two batches of inhibitor **3** were used in the present study, one batch had a purity of 95% (used in the Caco-2 assay) and the other 90% (used in the remaining experiments). Inhibitors **1** and **2** were diluted and stored in DMSO at a concentration of 20 mM, and inhibitor **3** at a concentration of 10 mM. All inhibitors were stored at −20 °C.

### Fluorescence anisotropy assay

A FA assay was used to determine the affinity of recombinant human galectin-3 and the three inhibitors in solution. The use of the FA assay for this purpose has previously been described in detail by Sörme *et al*.^49^. In short, increasing concentrations of galectins were first titered against a fixed concentration of the fluorescent saccharide probe 3,3′-dideoxy-3-[4-(fluorescein-5-yl-carbonylaminomethyl)-1H-1,2,3-triazol-1-yl]−3′-[4-(3-fluorophenyl)-1H-1,2,3-triazol-1-yl]-1,1′-sulfanediyl-di-β-d-galactopyranoside (4 nM) (described in Peterson *et al*.^28^), to obtain binding curves in which the anisotropy value increases from a value for probe free in solution (*A*_0_) to a value where all probe molecules are bound to galectin-3 (*A*_*max*_). Binding curves were constructed for each test condition used in this study (*i.e*. different buffer, temperature and pH, see below) (Fig. S4). To establish the dissociation constant (*K*_*d*_) values between the inhibitors and galectin-3, a competitive variant of the FA assay was utilized. In this study, increasing concentrations of the inhibitors were titered against fixed concentrations of galectin-3 and probe. By measuring the anisotropy values for the different inhibitor concentrations, together with the values for *A*_*max*_ and *A*_0_, the *K*_*d*_ values could be calculated according to the equations presented in Sörme *et al*.^49^.

The experiments were either performed in PBS (pH 7.2), in 50 mM phosphate buffer with 100 mM NaCl (pH 6, 7, or 8), or in 50 mM acetate buffer with 100 mM NaCl (pH 5). All buffers were supplemented with 0.1 µM BSA (to not lose any protein due to absorbance to plate surfaces). For the experiments performed in PBS (pH 7.2), a concentration of 20 nM galectin-3 was used (at room temperature and 4 °C). For the experiments done in phosphate/acetate buffer, galectin-3 was used at concentrations of 400 nM (pH 5), 100 nM (pH 6), or 50 nM (pH 7 and 8).

The emitted light from the fluorescent saccharide probes was measured using a PheraStarFS plate reader and PHERAstar Mars version 2.10 R3 software (BMG, Offenburg, Germany). The excitation wavelength used was 485 nm and the emission was read at 520 nm. The anisotropy values for each data point were read in duplicate wells of 96-well plates (at a total volume of 140 µl) 30 minutes after the addition of all solutions (for equilibrium to arise). *K*_*d*_ values were calculated as weighted mean values from inhibitor concentrations generating between 20–80% inhibition (where inhibition values of approximately 50% had the highest impact on the mean value).

### Cell culture

JIMT-1 cells were from the German Collection of Microorganisms and Cell Cultures (Braunschweig, Germany), and MCF-7 and HCC1937 cells from American Type Culture Collection (Manassas, VA, USA). JIMT-1 cells were maintained in DMEM/Ham’s F12 medium mixture (1:1), whereas MCF-7 and HCC1937 were maintained in RPMI-1640 medium. The cell culture medium of all three cell lines was supplemented with 10% FBS, 100 U/ml penicillin, 100 μg/ml streptomycin, 1 mM nonessential amino acids, and 10 µg/ml insulin, and for the HCC1937 cells additionally with 20 ng/ml epidermal growth factor. The cells were kept at 37 °C in a humidified incubator with 5% CO_2_ in air and were sub-cultured once per week. The CHOZN^®^ glutamine synthetase (GS)^−/−^ zinc finger nuclease-modified CHO cell line was purchased from Sigma-Aldrich (St. Louis, MO, USA), and cultured in EX-CELL^®^ chemically-defined serum-free medium for CHO cells supplemented with 4 mM l-glutamine as suspension cultures in 50 ml TPP^®^ TubeSpin bioreactor tubes (at 200 rpm, 36.5 °C and 5% CO_2_).

### Caco-2 paracellular permeability assay

The *P*_*app*_ values for the galectin-3 inhibitors, *i.e*. their ability to cross biological membranes, was determined in a standardized *in vitro* assay with the human colorectal carcinoma cell line Caco-2^34–36^. The experiments were performed either by Admescope Ltd (Typpitie 1, 90620 Oulu, Finland) or Chempartner Co., Ltd (DMPK Group, Shanghai ChemPartner Co., Ltd., Ha Lei Rd, Pudong, Shanghai, 201203, P.R. China). In brief, Caco-2 cells were seeded and grown to confluence on microporous polycarbonate membranes (pore diameter of 0.45 µm), whereupon they differentiate and form monolayers of polarized cells with tight junctions. The inhibitor to be tested was added to either the apical or basolateral side of the membrane and the concentration on the other side was measured (by liquid chromatography – tandem mass spectrometry) as a function of time, with the last sample taken after 90 or 120 minutes. *P*_*app*_ for each compound was calculated according to Equation 1:

1 $${P}_{{app}}=\frac{{\rm{d}}Q}{{\rm{d}}t}\,\cdot \,\frac{1}{A{C}_{0}}$$

where d*Q*/d*t* is the steady-state appearance rate of the compound, *A* is the surface area of the Caco-2 membrane and *C*_0_ is the initial concentration of the compound added^34^. All experiments were performed at a constant pH of 7.4 at 37 °C and all measurements were done in duplicates (n = 2). The integrity of the Caco-2 cell layers was monitored by trans-epithelial electrical resistance measurements before addition of compounds. In addition, reference compounds (high permeability: diclofenac or metoprolol, low permeability: atenolol, erythromycin, and digoxin) with known permeabilities were included in all experiments for validation and calibration.

### NHS-fluorescein labelling of galectin-3

A 15-fold excess of NHS-fluorescein dissolved in *N*,*N*-dimethylformamide was added to a 2 mg/ml of galectin-3 solution in 100 mM carbonate/bicarbonate buffer. The solution was mixed well and incubated for 1 hour at room temperature. Labeled galectin-3 was separated from the unreacted dye by a buffer change to PBS on a PD10 column. The degree of labeling was calculated according to the manufacturer’s instructions.

### Flow cytometry

CHOZN^®^ GS^−/−^ cells were cultured in suspension until reaching a density of 0.5–3 × 10^6^ cells/ml, harvested by centrifugation (500 × *g*), washed with 200 mM lactose in PBS (containing Mg^2+^ and Ca^2+^, at pH 7.4) to remove any endogenous galectins on the cell surfaces, and finally washed with PBS to remove excess lactose and re-suspended in PBS to obtain 2 × 10^6^ cells/ml. Aliquots of 200 µl (4 × 10^5^ cells) were added to the wells of a 96-well round-bottom plate, and kept on ice throughout the experiment. The cells were spun down in the plates, the PBS was removed, and to each well was added 100 μl PBS ± galectin inhibitors **1**, **2**, or **3** (at different concentrations) and 100 μl of PBS with NHS-fluorescein-labelled galectin-3 (0.5 µM). The cells were then incubated at 4 °C for one hour with gentle agitation. Fluorescence cytometry was performed on a LSRII instrument (Becton Dickson, San Jose, CA, USA). Fluorescence data was collected using logarithmic amplification on 30,000 light scatter-gated events (cell counts) and analyzed using FlowJo software. PI was added to a final concentration of 40–50 μg/ml just before cytometry to identify dead cells. Untreated CHO cells were used as controls in every experiment to normalize for variations in instrument sensitivity and sample handling. Negative controls were cells treated only with PBS ± galectin inhibitor. Dead cells as defined by high PI staining and cell aggregates, detected by a FSC-A *versus* FSC-H plot, were excluded from the analyses by gating and geometrical mean fluorescence intensity (GMFI) of the remaining sample cell populations was calculated.

### Treatment of cells with GPN and galectin-3 inhibitors

Cells were seeded in multi-well plates with sterile glass coverslips placed in each well and allowed to adhere to the cell culture surface for at least 24 hours. Then the cells were pre-treated with the different galectin-3 inhibitors at different concentrations (or DMSO as control) for 15 minutes or 24 hours followed by addition of the lysosomal damaging peptide GPN^42^ and incubated another 13 minutes, with the inhibitors still present. The 13-minute incubation with GPN and 15-minute pre-incubation with inhibitors were done in DMEM/Ham’s F12 medium with 15 mM HEPES buffer (without serum), whereas the 24-hour incubation with inhibitors were performed in fully supplemented DMEM/Ham’s F12 medium (as described under *Cell culture*). Control media contained DMSO concentrations of 0.1 or 0.5% (v/v) to match the DMSO concentration in the highest concentration of inhibitor used in the experiment (the DMSO concentration was the same in all wells in a given experiment, for the 24-hour pre-treatments the DSMO concentration did not exceed 0.1%). For the GPN treatment, DMSO at a concentration of 1.1–1.5% (v/v) was used as control.

### Detection of galectin-3 accumulation by immunocytochemistry

After treatment with GPN, the cells were fixed in 3.7% (v/v) formaldehyde in phosphate-buffered saline (PBS) for 15 minutes, permeabilized and blocked using 1% (v/v) TWEEN 20 and 5% (w/v) BSA in PBS for 30 minutes, then incubated with primary and secondary antibodies for one hour each (washed with PBS in between). Primary and secondary antibodies for galectin-3 staining were a rat anti-mouse galectin-3 IgG antibody^50,51^ (1:200) and a Alexa Fluor^®^ 594-conjugated goat anti-rat IgG antibody (1:600), respectively, and for LAMP-1 staining a rabbit anti-LAMP-1 IgG (1:600) and Alexa Fluor^®^ 488-conjugated goat anti-rabbit IgG (1:600), respectively. All antibodies were diluted in 0.05% TWEEN 20 and 5% BSA in PBS. The nuclei were stained using Hoechst 33342. The coverslips were mounted on glass slides using Mowiol^®^ 4–88 and left at 8 °C overnight before further examination. For imaging of the cells, a LSM-510 confocal laser scanning microscope (Carl Zeiss Microscopy GmbH, Oberkochen, Germany), equipped with a Hamamatsu R6357 photomultiplier (Hamamatsu Photonics K.K., Hamamatsu, Japan), was used. To excite the Alexa Fluor^®^ 594-conjugated antibody a 561 nm diode-pumped solid-state laser was used and a long-pass filter with a cut-off of 575 nm was used for the emission, whereas a 488 nm argon ion laser was used to excite the Alexa Fluor^®^ 488-conjugated antibody and a band-pass filter of 505–530 nm for the emission. For the excitation of Hoechst 33342, a 405 nm diode-pumped solid-state laser was used and a band-pass filter of 420–480 nm was used for the emission. After obtaining stacks of single optical planes (0.32 µm between each plane) in high magnification the cells were visualized using the software ZEN 2012 (black edition) version 8.0 (Carl Zeiss Microscopy GmbH). Maximum intensity projections of each picture obtained were analyzed with ImageJ 1.47 v (Rasband, W.S., ImageJ, U. S. National Institutes of Health, Bethesda, Maryland, USA) to quantify the number of galectin-3 dots and the number of nuclei in each picture. The quantification of particles (*i.e*. galectin-3 puncta/nucleus) was carried out using batch processing in the software, where a single macro (including parameters such as image thresholds, particle size and circularity) was used for all images obtained from a single experiment, this to reduce user bias and time consumption.

### MTT assay

The MTT (3-[4,5-dimethylthiazol-2-yl]−2,5-diphenyltetrazolium bromide) assays were performed as previously described^52^. In brief, cells were seeded in 96-well plates (6000 cells/well for MCF-7 and HCC1937 and 5000 cells for JIMT-1 in 180 μl fully-supplemented medium as described under *Cell culture*). The cells were incubated for 24 hours before addition of galectin-3 inhibitors. The inhibitors were diluted in PBS from their respective stock solution in DMSO (see *Galectin-3 inhibitors*) and used at final concentrations ranging from 0.04 to 40 μM. DMSO was adjusted to make the final DMSO concentration 0.2% for all inhibitor concentrations, including the control sample (no inhibitor). After 72 hours of treatment, 20 μl of 5 mg/ml MTT solution (in PBS) was added to each well and incubation continued for 1 hour at 37 °C. Thereafter, the MTT containing medium was removed and 100 μl of DMSO was added per well and the plates swirled gently for 10 minutes to dissolve the cells and the purple formazan product precipitate. Absorbance was monitored at 540 nm using a Labsystems iEMS Reader MF (Labsystems Oy, Helsinki, Finland) and the software DeltaSoft II v.4.14 (Biometallics Inc., Princeton, NJ, USA).

### Cell proliferation assay

JIMT-1 cells were seeded in 5 cm diameter Petri dishes at a density of 400 000 cells/Petri dish. Inhibitor **1** or **2** was added one day after seeding at the final concentration of 5 µM and control cells were treated with 0.2% (v/v) DMSO to match the DMSO concentration in inhibitor containing cultures. The cells were harvested by trypsinization and the number of cells was determined by counting in a hemocytometer (each day for a total of 6 days).

## Supplementary information

Additional data for inhibitor affinities, GPN-induced galectin-3 accumulation, inhibitor toxicity, and binding curves for fluorescent saccharide probe
