# Supplementary material for: Extracellular and intracellular small-molecule galectin-3 inhibitors
Source: Sci Rep. 2019 Feb 18;9:2186. doi: 10.1038/s41598-019-38497-8 (PMC6379368; doi:10.1038/s41598-019-38497-8)
Supplement: Supplementary file 1 — Additional data for inhibitor affinities, GPN-induced galectin-3 accumulation, inhibitor toxicity, and binding curves for fluorescent saccharide probe [file 41598_2019_38497_MOESM1_ESM.pdf]

Supplementary Information

Extracellular and intracellular small-molecule galectin-3 inhibitors

John Stegmayr[1,*], Fredrik Zetterberg[2], Michael C. Carlsson[3,#], Xiaoli Huang[4,†], Gunjan Sharma[1], Barbro Kahl-Knutson[1], Hans Schambye[2], Ulf J. Nilsson[5], Stina Oredsson[4], and Hakon Leffler[1,*]

From the [1]Department of Laboratory Medicine, Lund University, 22100 Lund, Sweden; [2]Galecto Biotech AB, 2200 Copenhagen N, Denmark; the [3]Department of Cellular and Molecular Medicine, University of Copenhagen, 2200 Copenhagen N, Denmark; the [4]Department of Biology, Lund University, 22100 Lund, Sweden; the [5]Department of Chemistry, Lund University, 22100 Lund, Sweden.

[#]Present address: Agilent Technologies Denmark ApS, 2600 Glostrup, Denmark

[†]Present address: Xintela AB, 22381 Lund, Sweden

[*]To whom correspondence should be addressed: John Stegmayr or Hakon Leffler: Box 117, Department of Laboratory Medicine, Lund University, 22100 Lund, Sweden; john.stegmayr@med.lu.se or hakon.leffler@med.lu.se; Tel. +46 (0)46 222 7579.

METHODS (not described in main article)

Cell culture

The AsPC-1, BxPc-3, MIA PaCa-2, and PanC-1 pancreatic cancer cell lines were purchased from the American Type Culture Collection (ATCC) (Manassas, VA, USA). AsPC-1 and BxPc-3 cell lines were cultured in RPMI-1640 medium, and MIA PaCa-2 and PanC-1 in Dulbecco's Modified Eagle Medium (DMEM)/Ham's F12 medium mixture (1:1). The medium of all four pancreatic cancer cell lines was supplemented with 10 % fetal bovine serum (FBS), 100 U/ml penicillin, and 100 μg/ml streptomycin. The L56Br-C1 breast cancer cell line was established at Lund University (Lund, Sweden)[1], and was cultured in RPMI-1640 medium supplemented with 10 % heat-inactivated FBS, 100 U/ml penicillin, 100 μg/ml streptomycin, 1 mM non-essential amino acids, and 10 μg/ml insulin. The MCF-10A normal-like breast cell line was obtained from ATCC and was cultured in the same medium mixture as L56Br-C1 with the addition of 20 ng/ml epidermal growth factor, 50 ng/ml cholera toxin, and 500 ng/ml hydrocortisone.

Fluorescence anisotropy assay

The affinity between inhibitor **1** and the carbohydrate recognition domain (CRD) of galectin-8C and between inhibitor **3** and the CRD of galectin-2, -4N, -4C, 8C, and -9C was determined using the fluorescence anisotropy assay described in the main article. Experimental conditions used for each galectin are listed below.

Galectin-2: experiments were performed in PBS supplemented with 0.1 μM bovine serum albumin at 20 °C with galectin-2 at 0.9 μM and the fluorescent probe 3,3'-dideoxy-3-[4-(fluorescein-5-yl-carbonylaminomethyl)-1H-1,2,3-triazol-1-yl]-3'-[4-(3,4,5-trifluorophenyl)-1H-1,2,3-triazol-1-yl]-1,1'-sulfanediyl-di-β-D-galactopyranoside at 0.02 μM[2].

Galectin-4N: experiments were performed in PBS at 20 °C with galectin-4N at 0.80 μM and the fluorescent probe 3,3'-dideoxy-3-[4-(fluorescein-5-yl-carbonylaminomethyl)-1H-1,2,3-triazol-1-yl]-3'-[4-(3-fluorophenyl)-1H-1,2,3-triazol-1-yl]-1,1'-sulfanediyl-di-β-D-galactopyranoside at 0.02 μM[2].

Galectin-4C: experiments were performed in PBS at 20 °C with galectin-4C at 0.50 μM and the fluorescent probe *N*-(2-acetamido-2-deoxy-α-D-galactopyranosyl-(1–3)-[α-L-fucopyranosyl-(1–2)]-β-D-galactopyranosyl-(1–4)-β-D-glucopyranosyl)-fluorescein-5-yl-carbonylaminobutanamide at 0.02 μM[3].

Galectin-8C: experiments were performed in PBS at 20 °C with galectin-8C at 0.60 μM and the fluorescent probe 3,3’-dideoxy-3-[4-(fluorescein-5-yl-carbonylaminomethyl)-1H-1,2,3-triazol-1-yl]-3’-(3,5-dimethoxybenzamido)-1,1’-sulfanediyl-di-β-D-galactopyranoside at 0.02 μM[4].

Galectin-9C: experiments were performed in PBS at 20 °C with galectin-9C at 1.00 μM and the fluorescent probe 3,3'-dideoxy-3-[4-(fluorescein-5-yl-carbonylaminomethyl)-1H-1,2,3-triazol-1-yl]-3'-[4-(3-fluorophenyl)-1H-1,2,3-triazol-1-yl]-1,1'-sulfanediyl-di-β-D-galactopyranoside at 0.02 μM[2].

SUPPLEMENTARY FIGURES

Figure S1

a

Inhibitor	K_d value (µM)								
	galectin-1	galectin-2	galectin-4N	galectin-4C	galectin-7	galectin-8N	galectin-8C	galectin-9N	galectin-9C
**1**	0.012±0.003[5]	>5[5]	0.17±0.029[5]	0.14±0.042[5]	1.9±0.38[5]	86±8.8[5]	**5.2±0.17**	0.68±0.34[5]	0.12±0.015[5]
**2**	3.7±0.15[6]	0.64±0.11[6]	2.9±0.40[6]	0.13±0.012[6]	31±3.7[6]	83±17[6]	11±1.9[6]	2.7±0.24[6]	2.4±0.41[6]
**3**	0.23±0.03[2]	**1.5±0.04**	**7.0±0.9**	**6.2±0.9**	5.4±0.9[7]	58±5[7]	**4.4±0.5**	1.1±0.3[7]	**1.0±0.1**

b

Inhibitor	K_d (galectin-x)/K_d (galectin-3)								
	galectin-1	galectin-2	galectin-4N	galectin-4C	galectin-7	galectin-8N	galectin-8C	galectin-9N	galectin-9C
**1**	5	2273	77	64	864	39091	2364	309	55
**2**	99	17	78	3	831	2225	295	72	64
**3**	6	42	196	173	151	1620	123	31	28

c

Inhibitor	K_d value (nM)	SEM
**1**	<1*	N/A
**2**	7.3	0.9
**3**	6.2	0.5

d

Inhibitor	pH 5		pH 6		pH 7		pH 8	
	K_d value (nM)	SEM	K_d value (nM)	SEM	K_d value (nM)	SEM	K_d value (nM)	SEM
**1**	100.2	14.6	10.7	1.8	3.6	1.4	2.6	0.3
**2**	830.9	55.5	156.5	11.2	46.4	5.1	47.4	5.1
**3**	777.7	68.0	163.8	9.4	39.2	3.9	37.0	2.9

Figure S1. Additional K_d values for the galectin inhibitors. (**a**) Summary of K_d values for inhibitors **1**, **2**, and **3** for galectin-1, -2, 4N, -4C, -7, -8N, -8C, -9N, and -9C as previously published (marked with the corresponding reference; Delaine *et al.*[5], Zetterberg *et al.*[6], Salameh *et al.*[7], and Peterson *et al.*[2]) or determined in this study (bold values). (**b**) The ratios of K_d (galectin-x)/K_d (galectin-3) for the three inhibitors (the K_d values for galectin-3 equals 0.0022, 0.0373 and 0.0358 µM for inhibitor **1**, **2**, and **3**, respectively (Fig. 1b)). (**c-d**) K_d values between inhibitors **1**, **2**, and **3** and galectin-3 at 4 °C (in PBS) and at different pH values (in phosphate/acetate buffers). The K_d values are presented as means from 4-30 measuring points (where the inhibitors generated 20-80 % inhibition of the galectin/probe interaction) from 3 independent experiments. * = under the detection limit of the assay.

Figure S2

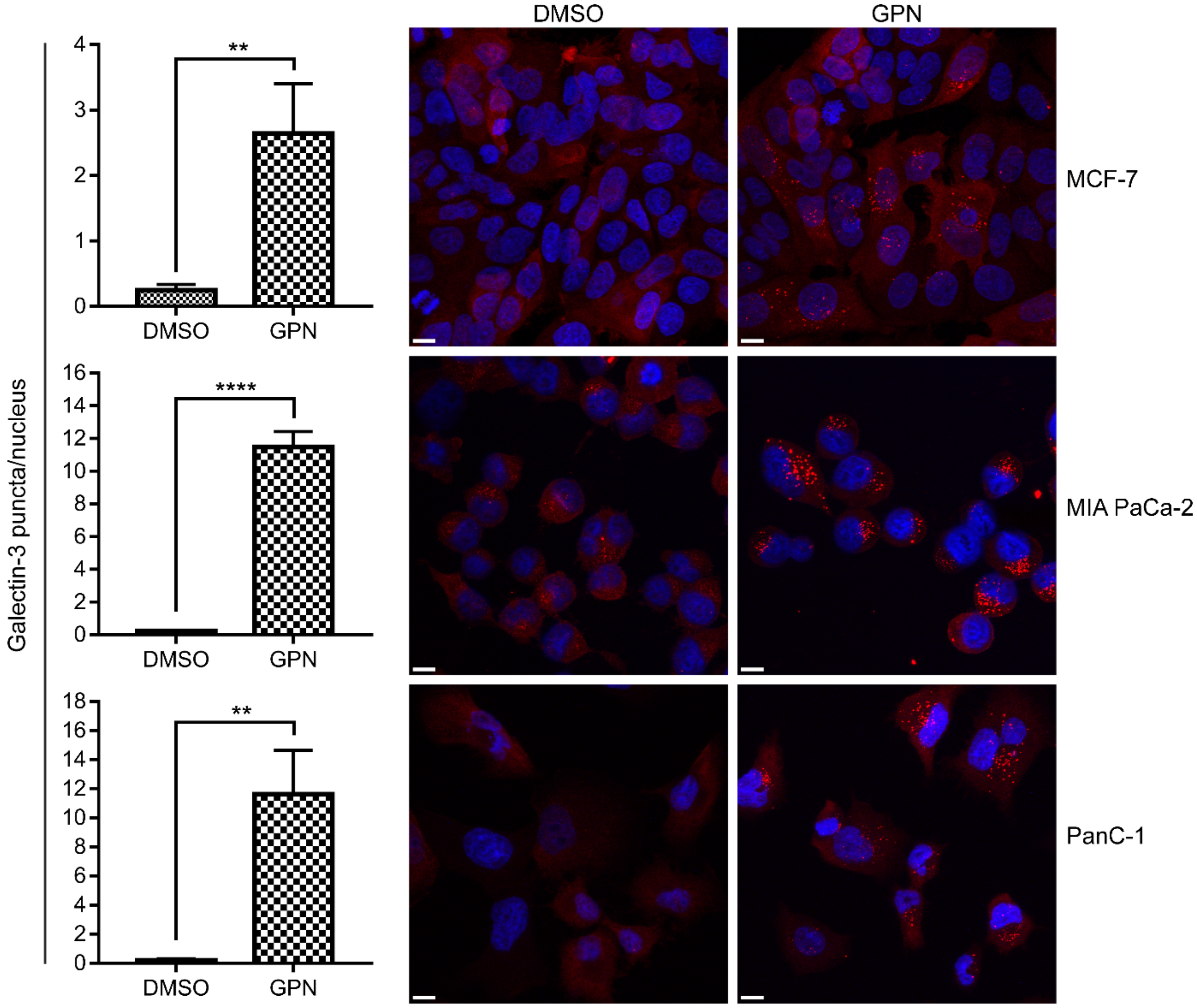

Figure S2. Induction of galectin-3 accumulation upon treatment with GPN in three additional cell lines. The number of galectin-3 puncta/nucleus was quantified in three cancer cell lines (MCF-7 breast cancer cells and MIA PaCa-2 and PanC-1 pancreatic cancer cells) treated with the lysosomal damaging agent, glycyl-L-phenylalanine 2-naphthylamide (GPN), at a concentration of 0.3 mM for 10-13 minutes, or 1 % dimethyl sulfoxide (DMSO) for the same amount of time, as control. The analysis was carried out in ImageJ after the *z*-stacks, obtained by confocal microscopy, were converted to two-dimensional images (maximum intensity projections). The result is presented as mean values ± SEM (6-10 confocal images from 2 independent cell cultures were analyzed for each mean value in the graphs). An unpaired two-tailed *t*-test was used to calculate statistical significance between the two treatment groups (** $P < 0.01$, **** $P < 0.0001$). Representative immunofluorescence images are displayed and treatment with GPN is seen to induce distinct galectin-3 puncta in the cytosolic space in the three cancer cell lines. Scale bars equal 10 µm.

Figure S3

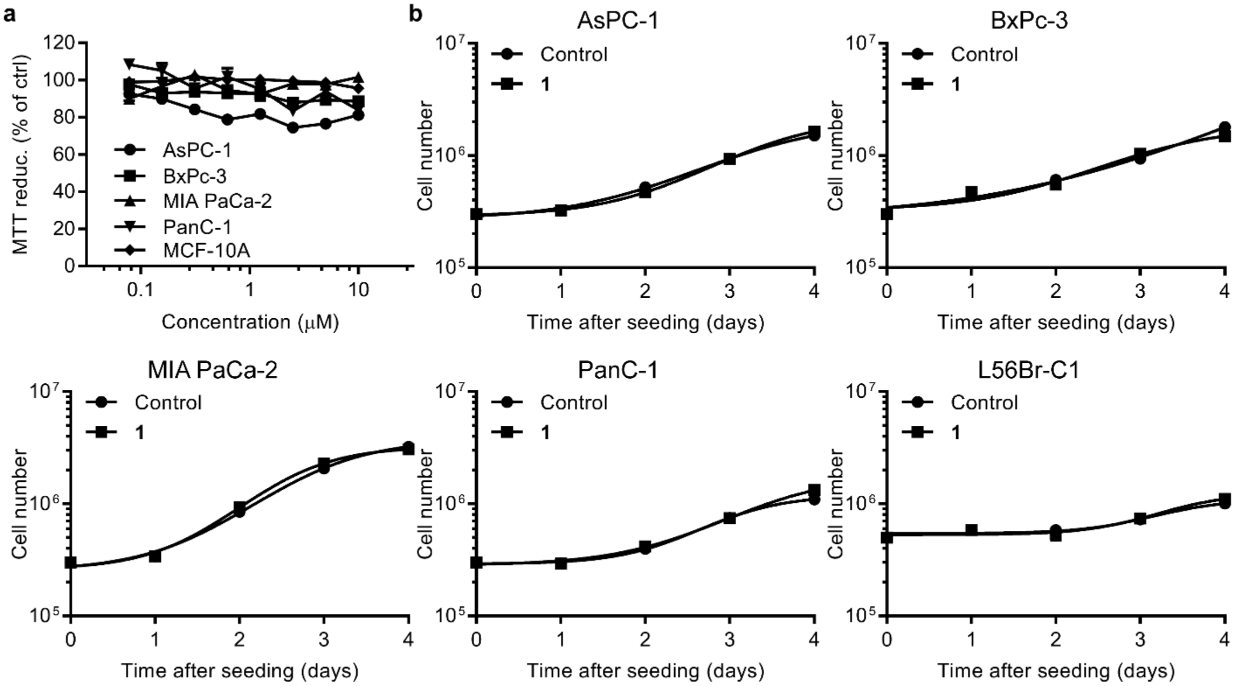

Figure S3. Low toxicity of galectin-3 inhibitor 1 in additional cell lines. (**a**) Inhibitor **1** was tested in an MTT assay for a treatment time of 72 hours (added 24 hours after cell seeding) in four pancreatic cancer cell lines and one normal-like cell line (MCF-10A). No dose-response relationship could be observed for inhibitor **1** in any of the cell lines. (**b**) Growth curves for the AsPC-1, BxPc-3, MIA PaCa-2, and PanC-1 pancreatic cancer cell lines and the breast cancer cell line L56Br-C1 grown in the absence or presence of inhibitor **1**. The cells were treated with a fixed concentration of 10 µM of inhibitor **1** or 0.1 % (v/v) DMSO (used as control) 24 hours after seeding. No effect on cell proliferation could be observed when **1** was compared to control, in accordance with the results presented in panel **a**. Results in both panel **a** and **b** are presented as mean values ± SEM (n=6 in panel **a** and n=3 in panel **b**).

Figure S4

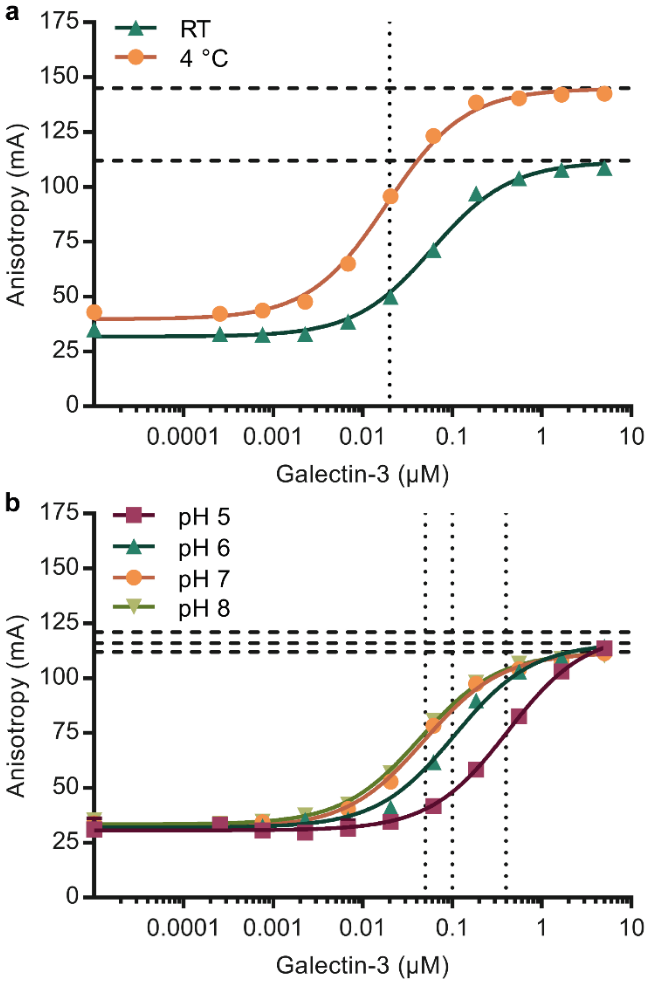

Figure S4. Binding curves for galectin-3 and a fluorescent saccharide probe. Recombinant human galectin-3 was titered against a fixed concentration of the fluorescent saccharide probe 3,3'-dideoxy-3-[4-(fluorescein-5-yl-carbonylaminomethyl)-1H-1,2,3-triazol-1-yl]-3'-[4-(3-fluorophenyl)-1H-1,2,3-triazol-1-yl]-1,1'- sulfanediyl-di-β-D-galactopyranoside (4 nM), whereupon the anisotropy was measured at different conditions, as described under *Methods* (main article). (**a**) Binding curves at room temperature and at 4 °C, 30 minutes after the addition of all reagents. Experiments were performed in phosphate buffered saline (**b**) Binding curves at the indicated pH values, 30 minutes after the addition of all reagents. Measurements were performed in acetate buffer (pH 5) or in phosphate buffer (pH 6, 7, and 8). The data points (panel **a** and **b**) are mean values obtained from duplicate wells of a black microtiter plate and the solid lines are best-fit curves, where $Y = A_0 + (A_{max} - A_0) * (X / (X + K_d))$. The dashed horizontal lines represent the calculated A_{max} values and the doted vertical lines highlight the galectin-3 concentrations used to establish the affinities for galectin-3 inhibitors **1**, **2**, and **3** in the competitive variant of the fluorescence anisotropy assay used in the main article.

REFERENCES

1 Johannsson, O. T. *et al.* Characterization of a novel breast carcinoma xenograft and cell line derived from a BRCA1 germ-line mutation carrier. *Lab. Invest.* **83**, 387-396 (2003).

2 Peterson, K. *et al.* Systematic Tuning of Fluoro-galectin-3 Interactions Provides Thiodigalactoside Derivatives with Single-Digit nM Affinity and High Selectivity. *J. Med. Chem.* **61**, 1164-1175, doi:10.1021/acs.jmedchem.7b01626 (2018).

3 Carlsson, S. *et al.* Affinity of galectin-8 and its carbohydrate recognition domains for ligands in solution and at the cell surface. *Glycobiology* **17**, 663-676 (2007).

4 Salomonsson, E. *et al.* Monovalent interactions of galectin-1. *Biochemistry* **49**, 9518-9532, doi:10.1021/bi1009584 (2010).

5 Delaine, T. *et al.* Galectin-3-Binding Glycomimetics that Strongly Reduce Bleomycin-Induced Lung Fibrosis and Modulate Intracellular Glycan Recognition. *ChemBioChem* **17**, 1759-1770, doi:10.1002/cbic.201600285 (2016).

6 Zetterberg, F. R. *et al.* Monosaccharide Derivatives with Low-Nanomolar Lectin Affinity and High Selectivity Based on Combined Fluorine-Amide, Phenyl-Arginine, Sulfur-pi, and Halogen Bond Interactions. *ChemMedChem* **13**, 133-137, doi:10.1002/cmdc.201700744 (2018).

7 Salameh, B. A., Cumpstey, I., Sundin, A., Leffler, H. & Nilsson, U. J. 1H-1,2,3-triazol-1-yl thiodigalactoside derivatives as high affinity galectin-3 inhibitors. *Bioorg. Med. Chem.* **18**, 5367-5378, doi:10.1016/j.bmc.2010.05.040 (2010).
